# Scraps

**Published:** 1887-09

**Authors:** 


### SCRAPS

PICKED UP BY THE ASSISTANT-EDITOR.

---

**The New Nomenclature.**—Georgie: "Do you know, Ethel, old Stokes had a perplexity fit the other day?" Ethel: "A perplexity fit? You mean a parallel stroke."

**Vaccination.**—According to the Chicago *Town Topics*, a story is going about that a certain "fayre ladye," who has made herself more than prominent in social and amateur theatrical circles, and in wearing an extremely low *corsage*, was anxious to be vaccinated before her recent departure for Europe. She consulted her physician on the subject, impressing upon him the absolute importance of performing the operation where it could not be seen, so that it would not interfere with her social and dramatic engagements. The doctor thought deeply on the all-important subject, and then advised her to swallow it.—*The Medical Press of Western New York.*

**Too many at once.**—It was the custom of the Roman physicians to visit their patients attended by all their pupils. Perhaps some hospital patients now-a-days have the same feelings that Martial had when he wrote the following epigram (V. ix.):

Languebam: sed tu comitatus protinus ad me
  Venisti centum, Symmache, discipulis.
Centum me tetigere manus, Aquilone gelatæ.
  Non habui febrem, Symmache: nunc habeo.

Here follow a couple of local attempts to render the above in English epigram:

(I.) TO DR. SYMMACHUS.

Hygiea frowned; thou sped'st with all thy train—
A hundred students—to relieve the pain:
Felt me all o'er; no fever racked my brow;
All Ætna's raging fires consume me now!

(II.) When out of sorts, to Symmachus I sent.
  A hundred pupils came along with him.
With chilling touch, and but on study bent,
  They start a fever through my every limb.

**Notes from Dublin.**—A correspondent of the *Irish Times* says that after the visit of the British Medical Association in 1867 "there was such an epidemic of hypochondriacs as frightened the whole community. Numbers of men, ay and women, aged and not, was readin' in the papers about all the diseases and maledictions that flesh is heir to, but doesn't always come into possession of any more than other heirs; and some of them took at once to bed, and fancied they had the same troubles themselves in elbow, heart, or heel." And he goes on to say: "I 'm afeard that now we 'll have the same over again. But I 'm the worst enemy they have in the world; and as for their drugs, I consider that what isn't pleasant to the taste isn't welcome to the intayrior. It 's a comfort the death-rate won't be heavy when the doctors is busy in other matters."

At the recent meeting a French doctor, returning thanks at one of the entertainments for the way in which the visitors had been received, said, "All Dooblin is von hospital boord." Of course this elicited some laughter, whereupon the guest said, "It is not faire; you do laugh at mine Englishe." But he was re-assured when one said to him, "You have unwittingly hit on a great truth. We have fifteen or sixteen hospitals in Dublin, and are of a verity over-hospitalled."—*Daily Express.*

"What is that medicine you are giving me, doctor?" "An emetic." "I won't take it: sorra use in it. The doctors in Dublin gave me two of them, and neither of 'em stayed two minutes on my stomach."—*The Hospital.*

**The Prince of Wales's Historic Glass of Norfolk Ale.**—Mr. John Bicker-Dyke writes to *The Daily Telegraph*:—"In your leader on 'The Curiosities of Ale and Beer,' you make mention of 'the historic glass of Norfolk ale for which the Prince of Wales asked after the turning crisis of his illness.' For the purposes of the chapter on the medicinal qualities of ale and beer I endeavoured, towards the end of last year, to obtain an accurate account of the circumstances surrounding that historic draught of barley wine. By Mr. Francis Knollys, to whom I first applied, I was referred to Sir William Gull, and permission was given me to publish whatever information on the subject he might afford. Sir William, I am sorry to find, looked upon the whole affair as a delusion on the part of the too credulous public. 'I regret,' he wrote, 'to have to destroy the illusion respecting the story of the bitter beer referred to in your letter. I can assure you the Prince never asked for bitter beer, and that it cannot be credited with any part of the successful issue of that important case.' As it cannot for a moment be supposed that the Prince of Wales's eminent medical adviser would draw any subtle distinction between bitter beer and other varieties of malt liquor in such a case as this, the ale was neither asked for nor given—at any rate, with the knowledge of Sir William Gull. It is curious, however, that the story, which appeared in several of the leading London journals, and which has for many years been generally accepted as accurate, should have remained so long uncontradicted. The incident illustrates the way in which history is made."

**Sympathy.**—Dr. Bowditch, of Boston, U.S.A., says:—"We are all members of a profession which, when regarded in its true light, above the plane of party strife and mere selfish gain, I regard as the finest and noblest of all, and the feeling grows stronger within me each year of practice. There is that in it far above the mere desire and ability to cure disease—that which can soothe all regrets for possible failure and disappointment in our daily work—I mean the power of human sympathy; the power which bids the young mother silently and gratefully press the hand that helped her in her hours of trial; the power that impels the dying man, at the very last, to turn to him who, though powerless to save, yet by a word, a look, a touch of the hand, gives strength and courage to one just passing to that 'undiscovered country from whose bourne no traveller returns.' In the midst of discord and disappointment let us keep this thought before us, and at the end perhaps we may be permitted to see our past life, as it were, stretched out before us, and feel that we have done our small share towards making our chosen profession what it should be—a blessing to all mankind." —*The Medical Age.*

Most medical men become aware, as they gain experience in their profession, that their popularity depends a good deal upon their manner in treating patients. But merely to study manners for the sake of popularity, is to risk their degenerating into "mannerism." Those who look upon their professsion as a Divine calling will remember to pray daily for sympathy; and, having prayed, they will go forth on their mission of healing without any studied effort to maintain manners, but rather trusting to the Great Physician to manifest His sympathy through them as He wills, and when He wills.—*The Hospital.*

